# Assisting the neurologist in diagnosis of CNS malignancies ‐ Current Possibilities and Limits of Cerebrospinal Fluid Cytology and Immunocytochemistry

**DOI:** 10.1002/brb3.805

**Published:** 2017-08-31

**Authors:** Jaroslava Dušková, Ondřej Sobek

**Affiliations:** ^1^ Institute of Pathology 1st Faculty of Medicine Charles University and Faculty General Hospital Prague Czech Republic; ^2^ Laboratory for CSF, Neuroimmunology, Pathology and Special Diagnostics Topelex Ltd Prague Czech Republic

**Keywords:** cerebrospinal fluid, cytology, immunocytochemistry, malignant cells

## Abstract

**Objectives:**

In tumorous impairment of CNS, cytological identification of the neoplastic cells in CSF frequently requires the use of ancillary techniques. Our methods are focused on identifying algorithms that increase the probability of identifying CSF malignant cells.

**Materials and Methods:**

A total of 1.272 CSF samples from patients with tumorous infiltration of CNS of nonhematologic origin along with 721 samples from patients with hematologic malignancies were analyzed in a complex setting including cytological and immunocytochemical investigations.

**Results and Discussion:**

In CSF diagnostics we are aware of the limited amount of sample combined frequently with neoplastic oligocytosis. Provided atypical, potentially malignant cells in CSF are found, further investigation(s) should maximize the *probability* of their identification—an appropriate cytological staining and immunocytochemical panel is to be applied. (i) In cases of known recent malignancy: immunoprofile of the recent neoplasm has been considered in immunocytochemical panel. (ii) In patients with a history of malignancy: The propensity to develop a new different malignancy must be taken into account. (iii) Atypical cells found in the CSF of a patient with a negative history of malignancy: Considering the most frequent clinically silent malignancies, stepwise immunocytochemistry is employed. Three milliliter of initial CSF sample represents the absolute minimum to start with.

**Conclusions:**

The steps of the laboratory activity targeted on malignancy in the CSF detection can be expected as follows: (i) The sample will be divided for both nonmorphology and cytopathology investigations. (ii) Basic stainings will triage the samples into those with no suspicion of malignancy and the remaining ones. (iii) Special stainings and stepwise immunocytochemistry will be performed in parallel with the nonmorphology investigations.

## 1 INTRODUCTION

Laboratory examination of cerebrospinal fluid is irreplaceable for diagnosing a wide spectrum of neurological diseases (Deisenhammer et al., 2006). Cytological examination is considered one of the substantial methods of CSF diagnostics (Sobek et al., 2012). A diagnostic asset of CSF cytology is evident in a large number of pathological affections of CNS: from inflammations in CNS compartment (incl. autoimmune), where cytological examination provides important information about immunocompetent cells in CSF (Sobek, Adam, Koudelková, Štourač, & Mareš, 2012; Zeman et al., 2001), to, for example, vascular damage of CNS, where cytological detection of erythrophages and macrophages containing hemosiderin or hematoidin is the method of choice to prove CT‐negative subarachnoid hemorrhage (SAH) (Sobek et al., 2012).

Tumorous impairment of CNS is the highest priority for the application of CSF cytology (Deisenhammer et al., 2006; Sobek et al., 2012). It is a minimally invasive diagnostic method, yet capable enough to ensure crucial information concerning not only the mere presence of tumorous cells in CSF but also their closer identification which is fundamental for consecutive therapy and for the patient's prognosis. Nevertheless, identification of the neoplastic cells is only partly achievable with cytomorphology and histochemical stainings (Glantz et al., 1998; Kaplan et al., 1990; Twijnstra et al., 1987; Wasserstrom et al., 1982). Ancillary techniques are now a part of guidelines and even routine investigations (Chamberlain et al., 2009; Chandra et al., 2009; Coakham et al., 1984; Weston et al., 2011).

The complex CSF investigation is oriented from the very beginning towards a precise diagnosis, enabling the clinician to start the most appropriate treatment for the patient. The subsequent text describing our laboratory algorithms in the identification of malignancies focuses on daily life diagnostic questions in different settings.

## 2 MATERIALS AND METHODS

During the years from 2010 to 2015, altogether 30.026 CSF samples were analyzed in the Laboratory for CSF, Neuroimmunology & Pathology, Topelex Ltd, Prague, of which 1.272 CSF samples were from patients with proven tumorous infiltration of CNS of nonhematologic origin. A total of 721 samples were obtained from patients with systemic hematologic malignancies and suspicion of CNS involvement, where in 349 cases malignant cells were found in CSF.

A complex setting of CSF investigations includes not only cytological but also biochemical, immunological, microbiological, and molecular‐genetic laboratory testing.

The immunocytology investigations are performed in cooperation with the Laboratory of Immunohistochemistry and Immunocytochemistry of the Institute of Pathology 1st Faculty of Medicine, Charles University, Prague.

### 2.1 Processing of cytological samples

In maximizing the diagnostic yield of the cells present, the CSF sample must be processed within 30 min (optimally) to a maximum of no more than 2 hr (some authors concede up to 3 hr (Sobek et al., 2012)) and maintained in‐between at 4°C. (To ensure this our laboratory has nonstop operating hours plus transport service.)

The common processing of the CSF sample for cytopathology investigation starts with cytocentrifuge (Cyto‐Tek® Sakura, up to 140×*g* for 5 min) or cytosedimentation slides stained according to May–Grünwald–Giemsa (MGG) and Hematoxylin‐Eosin (H&E).

The classical techniques of cytopathology laboratories (alcian blue, mucikarmine, PAS, oil red) are cheap and can be very helpful if purposefully applied. Many of these slides, especially the unmounted MGG, are subsequently usable for immunocytochemistry provided the cells in question are found to be present.

Based on the conventional cytological investigation, if there are cellular elements morphologically suspicious of malignant character found, then it is followed up with immunocytochemical (ICC) identification. The number of microscopic slides that are available for ICC investigations are unfortunately often limited by total sample volume, but optimally 4–5 further slides for ICC should be prepared.

### 2.2 ICC Methodology

- As a fixative, pure methanol is used for 5 min p.a. (according to the bottle min. 99,8%). After that the excess methanol is poured off and slides are air‐dried again. Till the phase of actual ICC, the microscopic slides are refrigerated at 2–8°C.
- Before proceeding with ICC, the microscopic slides are rehydrated for 5 min in distilled water.
- The next step is heat‐induced epitope retrieval in pH 6,0 or pH 9,0 buffer—depending on the subsequently used antibody—at 95–97°C in water bath for 20 min.
- The slides are then cooled down together with the detection buffer in cold water for 5 min and after that the cooling process continues with just the separate slides in a wash buffer for another 5 min (EnVision FLEX Wash Buffer manufacturer DAKO—thereinafter just the buffer).
- Inhibition of endogenous peroxidase in 3% hydrogen peroxide solution for 20 min.
- Thorough rinse for 3 times 5 min with buffer, application of a primary specific antibody. Concentrated antibodies are diluted according to the ratio recommended by the manufacturer, using dilution solution Primary Antibody Dilutent (manufacturer Diagnostic BioSystems). Incubation with primary antibody takes place in a moist chamber at room temperature for 30 min.
- Thorough rinse with buffer is followed with a 5 min bath in buffer in a cuvette.
- Then an application of a detection enzyme EnVision FLEX/HRP follows (manufacturer DAKO), and incubation in a moist chamber at room temperature for 30 min.
- Then the slides are rinsed with buffer and washed in distilled water for 5 min.
- The next step is an application of chromogen EnVision FLEX Substrate Working Solution (manufacturer DAKO). Incubation for 5 min, followed by a rinse with distilled water.
- Staining of the nuclei of cells with Hematoxylin (manufacturer DiaPath), for 1–2 min is followed with a rinse in “spring water” for 5 min.

### 2.3 Methodology notes

There is an exception for slides with antibodies which do not require heat‐induced epitope retrieval. These slides start with rehydratation first and continue straight with inhibition of endogenous peroxidase. Usually these antibodies are applied for longer period of time (overnight) at 2–8°C. Further processing steps are the same.

There is also an exception in fixation process for slides with antibodies which do not tolerate alcohol fixation (e.g., S100, GCDFP‐15, etc.)—in those cases formalin is used. Fixed slides are refrigerated, or may be kept at room temperature provided that ICC is done immediately. In the framework of this study there were no other medical examinations in patients or volunteers carried out, only anonymous clinical data were employed.

From each patient whose medical examination results are anonymously used in this manuscript, the authors have an informed consent for research use at their disposal.

## 3 RESULTS AND DISCUSSION

### 3.1 What are the clinical situations requiring identification of neoplastic cells in the CSF?

Generally, there are three different initial arrangements:

1. Recently treated known malignancy. CNS (incl. meningeal) impairment supposed.
2. History of malignancy treated/cured. Signs of CNS impairment present.
3. No history of recent or past malignancy, unclear neurological symptoms.

To achieve the solution and precise diagnosis, the limited amount of sample—usually no more than 8 ml—is divided into parts for chemical, microbiology, immunology, and cytopathology analyses. Many classical texts and articles dealing with the CSF diagnostics (Chandra et al., 2009; De May, 1996; Weston et al., 2011) stress the initial need of 3–5 ml CSF minimally to perform a valid investigation along with the possible requirements for an additional sample. Considering the fact that the declared minimal invasiveness of the spinal tap is a relative entity, and the sample received in the laboratory represents an extremely valuable source of information, all measures contributing to the optimum utilization should be employed to achieve the available diagnostic maximum.

### 3.2 How do we approach the three clinical situations mentioned above?

In all three settings, the more introductory information provided, the higher the probability of a precise interpretation.

- Patients' data: Age and gender are always available, race and country of origin (or travelers' history) may be of importance.
- Clinical symptoms (focal neurologic signs, intracranial hypertension, meningeal irritation, inflammatory features).
- Nonmorphological investigation results (biochemistry, immunology, microbiology, etc.).
- Morphology findings:
  1 Macromorphology—imaging (CT, MRI, arteriography, etc.).
  2 Micromorphology—in case of positive malignancy history, providing both this history and the previous histopathology results including the immunoprofile of the malignancy reported can greatly enhance the efficacy of the confirmatory investigations.

There are of course limits to be considered when designing the investigation steps and in interpreting the results:

- Even when an intracranial malignancy is present, the neoplastic cells are not necessarily present in the CSF.
- The past or present malignancy and its treatment and/or concomitant diagnoses can be accompanied with nonmalignant atypical cells.

#### 3.2.1 Evaluating the CSF finding

When evaluating the CSF finding, different results can occur. In the case of no suspicious cells present, a negative report is issued and the neurologist has to decide whether the provided diagnosis is compatible with the patient's status. When the negative result does not match the clinical course of the disease and no reliable explanation for the symptoms is available, false negativity of the first sample must be considered. The investigation should continue, especially if other nonmorphology investigations suggest the possibility of malignancy.

Provided atypical cells are found, further investigation(s) should maximize the *probability* of their identification using the best strategy available. While evaluating the morphology and possibly also the immunocytochemical results of CSF slides with atypical cells, many factors are to be considered, mainly:

- reactive and regressive changes in cells.
- cytocentrifuge artifacts—cell crowding or disaggregation, irregular nuclear contours, conspicuous nucleoli, cytoplasm fragility, and vacuolization.

These cytomorphology features overlap largely with those used for malignant cell identification. We start with the broadest general examination possible using the proper fixation while protecting and saving the sample from the very first step (see Materials & Methods section).

Cells in body fluids tend to degrade and lose their immunoreactivity. This is especially true for the hypotonic environment of cerebrospinal fluid. Therefore, emphasis is placed on fast processing and corresponding fixation. The most common protocols use 100% methanol or 4% buffered paraformaldehyde. Such fixed preparations generally retain immunoreactivity for several days when refrigerated. Longer storage (weeks or months) may weaken immunoreactivity (Fowler & Lachar, 2008).

Standards for immunocytochemical procedures develop more slowly than for immunohistochemical procedures. This is undoubtedly due to the obstacles in ensuring the necessary controls. Air‐dried preparations are widely used in cytology of aspirations of solid masses. The smears are created at the sampling point, air‐dried, and thus delivered to the laboratory. They are suitable especially for May–Grünwald–Giemsa staining. Polychrome staining methods (Papanicolaou) require wet fixation. Rehydration of air‐dried smears has been described in many articles. A cross‐sectional study of air‐dried smears versus wet fixation was published by Rupinder et al. (2013).

Cytological specimens that are primarily liquid in nature, cavity fluids including CSFs, are usually processed in the laboratory. Air‐dried preparations preserve immunoreactivity only for some, usually cytoplasmic antigens. The quality of membrane antigen manifestation can be impaired as demonstrated by Pinheiro et al. (2015).

Being conscious of the preciousness of the CSF sample, we use a proven methanol fixation protocol and refrigerate the reserve preparations. Recently, however, we also test the protocol recommended by Pinheiro et al. (2015). Coating of the preparation with polyethylene glycol after immediate methanol fixation allows storage of the preparations at room temperature. Rehydration can improve the immunoreactivity of air‐dried smears (Shidham et al., 2000) but air‐drying fixation represents an accepted version for immunocytochemistry (Fulciniti et al., 2008; Knoepp et al., 2013).

A short air‐drying step lasting 5 min prior to the 100% methanol fixation was in our protocol since it decreased cell loss in the subsequent procedures. All the illustration cases are processed this way. Nevertheless, our experience is similar to many other investigators in the necessity to adjust the protocols to the antibody in question and to the cytology material available (Sauter et al., 2016).

After the possibly malignant cells have been found, an appropriate immunocytochemical (mini) panel is to be designed (see Table 1).

**Table 1:** Immunocytochemistry minipanel in patients with negative history and suspicious malignant cells in the CSF

Antibody (Producer/Cat. Nr.)	Neoplasm Detected	Antigen Retrieval	Incubation	Dilution
CK, clone AE1/AE3 (Dako,/IS053)	Carcinomas	Citrate buffer bath 98°C	30 min	RTU
CD 45/LCA/, clone 2B11 + PD7/26 (Dako, IS751)	Lymhpoid or myeloid cells both neoplastic and non‐neoplastic	Citrate buffer bath 98°C	30 min	RTU
GFAP, clone 6F2 (Dako, IS524)	Glial tumors	Target retrieval solution High pH 9.0 98°C	30 min	RTU
Melanosome, clone HMB 45 (Dako, M 0634)	Melanoma	Incubation in the cold overnight		1:50
S100 protein (Dako, Z 0311)	Primary brain tumors, melanoma	Citrate buffer bath 98°C	30 min	1:400
Melan‐A, clone A103 (Dako, IS633)	Melanoma	Target retrieval solution High pH 9.0 98°C	30 min	RTU

RTU, Ready To Use Antibody.

In cytological examinations of other body fluids, the cost effectiveness of the diagnostic procedure is usually considered and stepwise diagnostic algorithms are employed. In CSF diagnostics we are aware of the limited amount of sample combined frequently with neoplastic oligocytosis. Irrespective of the exponential increase in antibodies available for routine diagnostics, neither individual antibodies, nor panels provide 100% sensitivity and specificity of detection. Nevertheless, prudent choice of markers can help. In neoplastic oligocytosis, it can be a helpful strategy to recycle the same scarce cells negative in certain markers to test them for others. It is advisable to ensure the microphotography documentation of the cells explored—any further laboratory test can cause their loss due to detachment from the slide.

### 3.3 Algorithms employed in our laboratory in relation to the three clinical situations outlined above

#### 3.3.1 Ad (1) Recently treated malignancy

**Figure 1:**
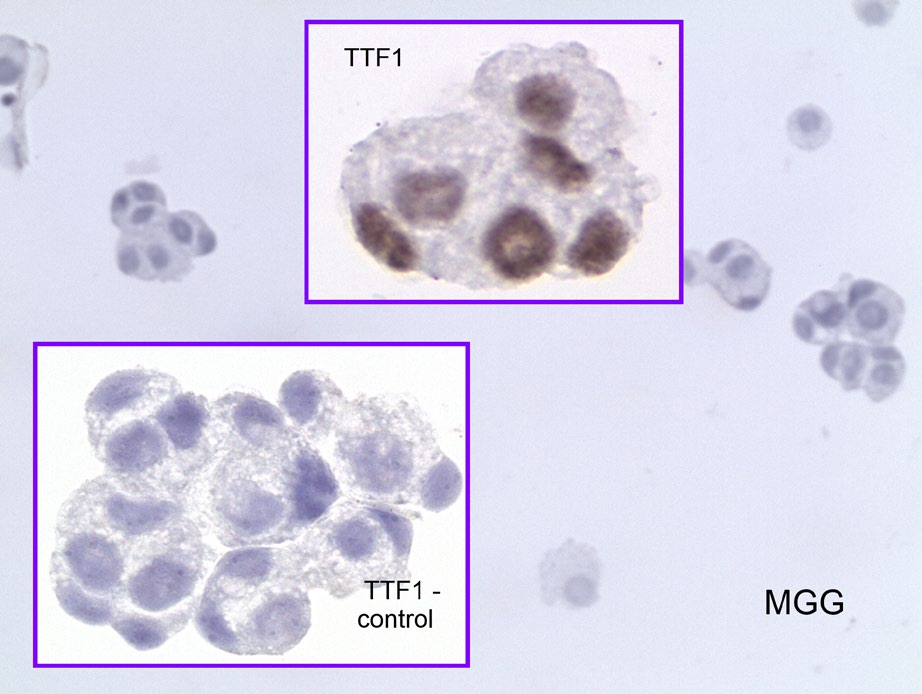
Male 73 years old. Recent lung carcinoma diagnosis TTF1 positive. CSF: pleocytosis with cohesive groups of cells with carcinoma features. TTF1—nuclear positivity. Control (primary antibody omitted) Nuclei negative. (TTF1—Thyroid Transcription Factor; CSF—cerebrospinal fluid). Magnification—objective: MGG 20×, TTF1 40×

With a known diagnosis we always try to get the previous biopsy and immunoprofile. Comparison of the morphology (considering at the same time the changes in solid neoplastic cells present in the liquid environment) is a rule of thumb here (violated frequently by young inexperienced consulting colleagues) (Figure 1). The neoplastic markers positive in the immunohistochemical investigation of the primary neoplasms are tested preferably on the suspicious CSF sample. When interpreting such a result, both the false negativity and false positivity must be taken into consideration. The false negativities occur due to fixation or simply due to the fact that the CSF contains a subset of cells that did not test positive in the primary. A false positivity manifests frequently on morphologically altered cells—a very critical evaluation of the cells preservation and positivity location considering the status of the whole preparation can help to prevent this misinterpretation.

#### 3.3.2 Ad (2) History of malignancy

**Figure 2:**
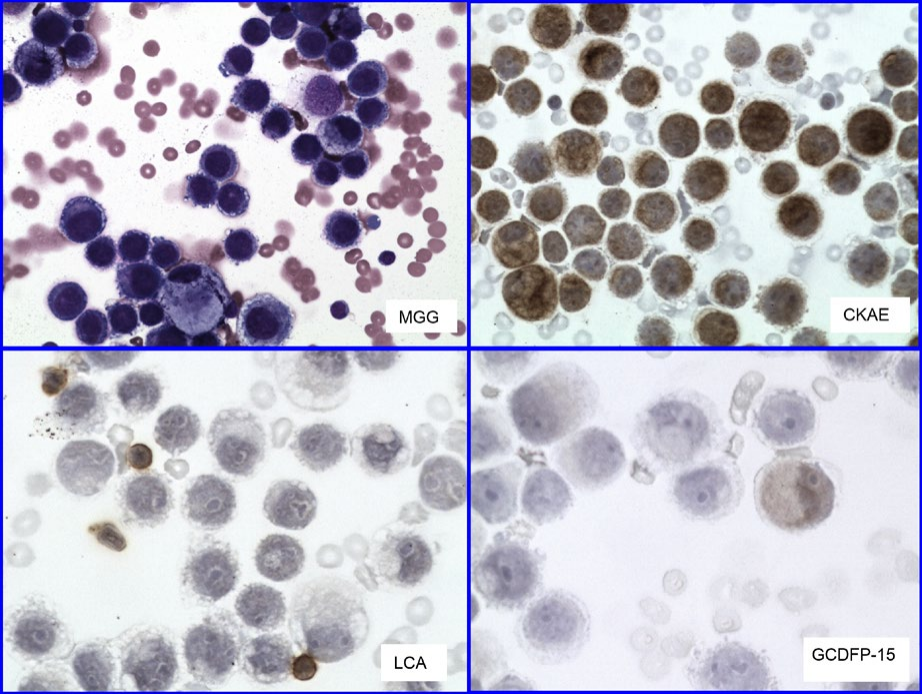
Female 53 years old. No history except ICD diagnostic code R 298 (Other and nonspecified symptoms of central nervous system, including meningismus) provided. Neoplastic pleocytosis (MGG) proved CKAE positive. LCA only small companying lymphocytes. History of mammary carcinoma traced back. Mammary marker GCDFP‐15 exhibited week cytoplasmic positivity in some cells. (ICD—International Classification of Diseases; MGG—May–Grünwald–Giemsa; CKAE—Cytokeratins AE1/AE3 cocktail; LCA—Leukocyte Common Antigen; GCDFP‐15—Gross Cystic Disease Fluid Protein 15kD). Magnification—objective: MGG and CKAE 20×, LCA and GCDFP‐15 40×

Recently, two features have become obvious with advanced therapy of malignancies (Figure 2). First, recurrences occur after longer and longer periods in many tumors, other than those known in the past to behave this way, for example, melanoma. Breast or kidney carcinomas recur after a period previously considered improbable for such a turn in the disease.

Second, (subsequent) primaries become more and more common due to the immunocompromising effects of the efficient oncology therapy. The practical impact is that the long known and diagnosed malignancy that was considered cured can become the source of recent disease. This should be exploited diagnostically and ruled out as described before. To trace back a previous biopsy is not always an easy task. Malignancies treated successfully years ago may escape inclusion into recent clinical data. A second primary in a known past malignancy represents an important differential diagnosis.

#### 3.3.3 Ad (3) Suspicious malignant cells found in the CSF sample of a patient with entirely negative history of tumorous disease

**Figure 3:**
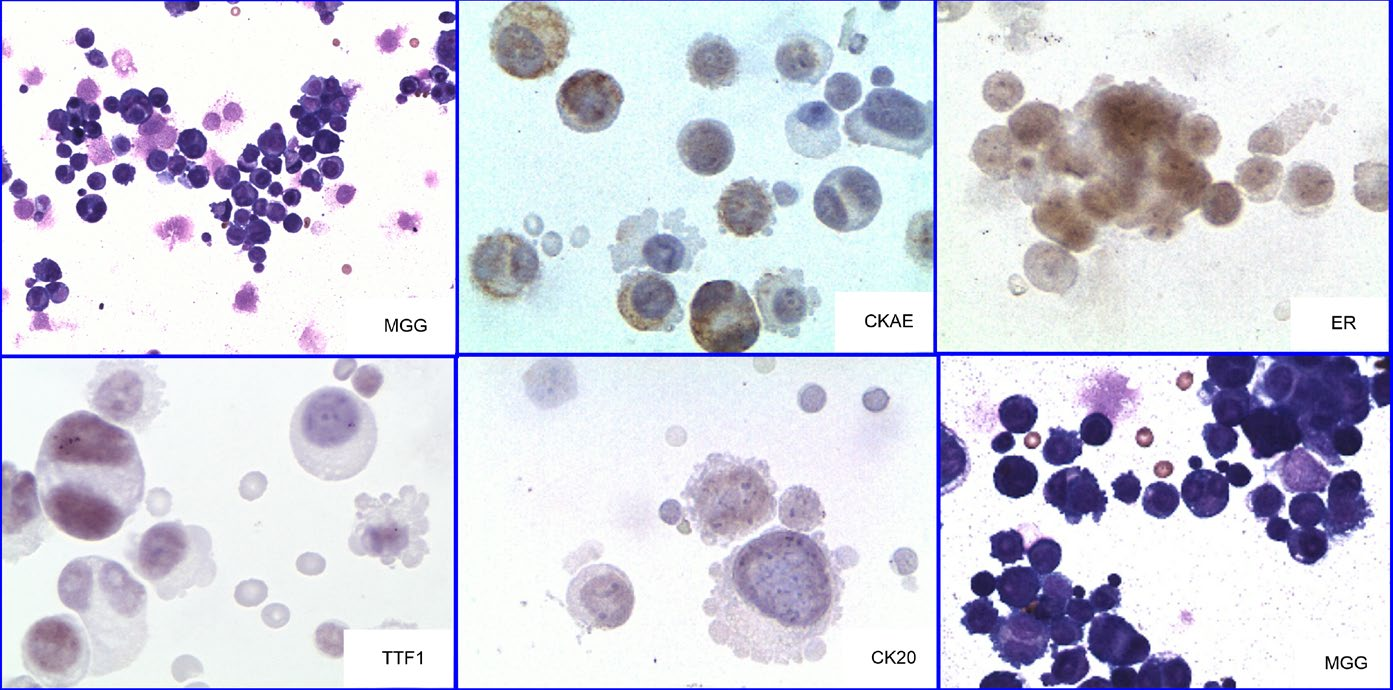
Female 55 years old. No malignancy history. Protracted instability. Arterial hypertension decompensated. Cervicocranial syndrome at first without meningeal irritation, recently vomitus and eye movement disorders. Impairment during the last fortnight. Cerebrospinal fluid exhibited neoplastic pleocytosis. Cytokeratins (CKAE) positive. Estrogen receptors positive. TTF‐1 weak equivocal positivity. Cytokeratin CK 20 negative. No further material for extension of the panel. Follow‐up: died one month later. Autopsy: generalization of ductal breast carcinoma clinically latent for a long time. (MGG—May–Grünwald–Giemsa; CKAE—Cytokeratins AE1/AE3 coctail; ER—estrogen receptor; TTF1—Thyroid Transcription Factor;) Magnification—objective: MGG10× and 20×, CKAE and ER 20×, TTF1 and CK20 40×

The broadest differential diagnostic judgments together with the limited sample represent the most challenging reality in these situations (Figure 3). Three milliliter of initial CSF sample represents the absolute minimum to start with, considering the statistical data of the most frequent clinically silent malignancies (lung, breast, kidney, pancreas,… melanoma) and verifying in parallel the negativity of the patients' malignancy history. The starting histogenetic panel identifying carcinomas, melanoma, and lymphoma using CKAE, Melan A, and LCA antibodies can be subsequently followed with a specifying step – organ‐related (rather than specific) markers like TTF1 (lung and thyroid), hormonal receptors, and GCDFP ‐15 (breast, the last one also salivary and sweat gland carcinoma) in concordance with the continuing clinically oriented investigation. During this period an additional sample from repeated spinal tap is a fully appropriate demand, provided the identification of the source of malignant cells remains hidden.

The quantitative cell content of the specimen plays an important role in the interpretation of the immunocytological results. The standardized criteria applied for investigations of other body fluids in accredited laboratories are more easily met in a CSF sample with neoplastic pleocytosis. Nevertheless, it would be unethical not to interpret, albeit with all prudency, the neoplastic oligocytosis as well. This question is addressed in previous studies (Fowler & Lachar, 2008). In fact, reporting of rare or even isolated suspicious cells is anchored in standardized reporting recommendations such as the Bethesda system for reporting cervical cytology or the Bethesda system for reporting thyroid FNAB (Ali & Cibas, 2010; Nayar & Wilbur, 2015).

The diagnostic success is more frequently achieved in solid metastatic malignancies (representing 60% of all malignancies in the CSF). In hematology (30% of all malignancies in the CSF) flow cytometry has been confirmed as a method of choice superior to immunocytopathology by many studies (Chamberlain et al., 2009; Chandra et al., 2009; Kaplan et al., 1990; Weston et al., 2011). Nevertheless, it faces frequently the same problem of sample volume. Primary malignancies in the brain represent only 10%; as they are usually deeply located they may frequently exhibit negative CSF.

In investigating the CSF sample with the neoplastic oligocytosis, recycling of the preserved cells can be successfully used. Both formulation of such results and the clinical interpretation and application must be done with strict awareness of the quantitative limit and thus possibly limited validity. It has been described thoroughly and also beautifully illustrated by Perske et al. that no single morphological parameter is sufficient to detect neoplastic lymphocytes. Taking into account a combination of cell size and irregular shape of cell and nucleus, however, may improve the diagnostic accuracy (Perske, Nagel, Nagel, & Strik, 2011). Meningeosis neoplastica occurs in 5%–10% of the most frequent malignancies, lung and breast cancer, melanoma, and diffuse large B‐cell lymphoma. The diagnosis opens the way to the intrathecal therapy (Strik & Prömmel, 2010).

Immunocytochemistry serves recently the “theranostic” field—detecting the protein exprimed as a result of translocations, fusions, and amplifications of genes and thus saving costs of molecular methods, being a cheaper alternative or the first step in triage. The detected protein informs about the responsiveness to the therapy, or prognosis (Chivukula & Dabbs, 2010; Swanson, 2015).

In connection with the economic and time factors of diagnostics, the question often arises as to whether immunocytochemical investigations should be performed in a patient with recent cancer and neoplastic meningeosis that is obvious in classical stainings. This is undoubtedly a more straightforward diagnostic situation, but there are several good reasons why reduced immunocytochemical testing should be performed: the first reason being that the metastatic cells in fluid exhibit changes in morphology.

In the case of a known immune profile of recent malignancy, subpopulations with certain immunocytochemical characteristics may be identified. Confirmed expression of certain markers, such as hormonal receptors, may influence the choice of therapy. Finally, with the current success of anticancer treatment, we are increasingly experiencing patients who have a history of two or even three malignancy processes. Clinical assignment then requires information on which of the previously proven processes is responsible for a positive finding in cerebrospinal fluid.

## 4 CONCLUSION

Close cooperation between the diagnostic laboratory and clinicians will result in a quick and accurate diagnosis, enabling appropriate treatment.

Necessary conditions on the laboratory side are:

- continuous availability (nonstop service)
- equipment with modern technologies.
- staffing with experienced specialists.

On the part of clinical specialists it is necessary to ensure in particular:

- timely delivery of the sample in a nondegraded quality and quantity required for diagnosis (in tumor diagnostics the initial minimum for cytopathology only requires 3–5 ml, additional sample requirements are not excluded)
- detailed information
  ○ on current clinical manifestations
  o results of imaging studies
  ○ anamnestic data, in particular about the current and past malignancies diagnosed and treated

When these introductory conditions are fulfilled, the steps in the laboratory activity targeted on malignancy in the CSF detection can be expected as follows:

- The sample will be divided for both nonmorphology and cytopathology investigations
- Basic stainings will triage the samples into those with no suspicion of malignancy and the remaining ones
- Special stainings and stepwise immunocytochemistry will be performed in parallel with the nonmorphology investigations
- The final report will be signed out in the shortest time possible—ideally on the same day as the completion of the cytological, resp. immunocytochemical investigation.

Cooperation and close contact among the team members throughout the entire process contributes greatly both to the desired results as well as to the greatest benefit for the patient.

## CONFLICT OF INTEREST

None declared.
